# Universal Plasma Jet for Droplet Manipulation on a PDMS Surface towards Wall-Less Scaffolds

**DOI:** 10.3390/polym13081321

**Published:** 2021-04-17

**Authors:** Cheng-Yun Peng, Chia-Hung Dylan Tsai

**Affiliations:** Department of Mechanical Engineering, National Yang Ming Chiao Tung University, Hsinchu 30010, Taiwan; heyhey.me04@nctu.edu.tw

**Keywords:** droplet manipulation, plasma jet, surface modification

## Abstract

Droplet manipulation is important in the fields of engineering, biology, chemistry, and medicine. Many techniques, such as electrowetting and magnetic actuation, have been developed for droplet manipulation. However, the fabrication of the manipulation platform often takes a long time and requires well-trained skills. Here we proposed a novel method that can directly generate and manipulate droplets on a polymeric surface using a universal plasma jet. One of its greatest advantages is that the jet can tremendously reduce the time for the platform fabrication while it can still perform stable droplet manipulation with controllable droplet size and motion. There are two steps for the proposed method. First, the universal plasma jet is set in plasma mode for modifying the manipulation path for droplets. Second, the jet is switched to air-jet mode for droplet generation and manipulation. The jetted air separates and pushes droplets along the plasma-treated path for droplet generation and manipulation. According to the experimental results, the size of the droplet can be controlled by the treatment time in the first step, i.e., a shorter treatment time of plasma results in a smaller size of the droplet, and vice versa. The largest and the smallest sizes of the generated droplets in the results are about 6 µL and 0.1 µL, respectively. Infrared spectra of absorption on the PDMS surfaces with and without the plasma treatment are investigated by Fourier-transform infrared spectroscopy. Tests of generating and mixing two droplets on a PDMS surface are successfully achieved. The aging effect of plasma treatment for the proposed method is also discussed. The proposed method provides a simple, fast, and low-cost way to generate and manipulate droplets on a polymeric surface. The method is expected to be applied to droplet-based cell culture by manipulating droplets encapsulating living cells and towards wall-less scaffolds on a polymeric surface.

## 1. Introduction

Droplet manipulation is an important technique for engineering, biomedical and chemical research because it not only drastically reduces the volume of samples but also significantly improves the efficiency of the tests [1,2,3]. There are different approaches for manipulating droplets, such as electrowetting-on-dielectric (EWOD) [4,5], magnetic manipulation [6,7,8], acoustic manipulation [9,10], dielectrophoretic (DEP) [11,12], optoelectrowetting [13,14] etc. For example, Zhang et al. [15] developed a microfluidic-based nozzle for fabricating microspheres from high-viscosity bioink. Du et al. [16] used a tapered capillary connected with a syringe pump for screening cell-based drug combination on a droplet-based microfluidic system. Droplet with biocompatible, biodegradable polymers can be used as scaffolds for cell cultivation [17,18]. For example, Um et al. [19] developed a continuous generation of hydrogel beads with encapsulation of biological materials for tissue engineering. Li et al. [20] used aligned fibers in polyglycerol sebacate (PGS) and polyvinyl alcohol (PVA) scaffolds for directing cell alignment.

Patterned-wettability technology is important for manipulating fluid on a surface and applications [21,22,23,24]. Among different patterning techniques, atmospheric-pressure plasma jet (APPJ) has been demonstrated as an efficient tool for patterned modifications on different surfaces, such as polycarbonate (PC) [25], polypropylene (PP) [26], polyethylene terephthalate (PET) [27], polymethyl methacrylate (PMMA) [28], polydimethylsiloxane (PDMS) [29] and even metallic surfaces [30,31,32]. The surface modification with an APPJ has been applied to different applications. For example, Thomas et al. [33] combined plasma printing and rotogravure printing for increasing the printing resolution with nano inks. Liu et al. [34] treated superhydrophobic aluminum surface with an APPJ for controlling water adhesion on the surface. Wu et al. [35] applied hydroxyl radicals from an APPJ to inhibit P. aeruginosa and C. albicans for treating green nail syndrome. Cheng et al. [36] demonstrated promising results of using argon APPJ for healing wound on streptozotocin-induced diabetic rats. Patelli et al. [37] processed implantable devices by an APPJ for specific surface morphologies and chemistries to interact with living systems. Lin et al. [38] added a small amount of oxygen into the working gas of an argon APPJ and found improved efficiency on sterilizing bacteria and endospores. In our prior studies, we have applied APPJ for plotting wall-less microfluidic channels on a PDMS surface and investigated its characteristics [39,40]. Here in this work, we proposed a method that uses APPJ to generate and manipulate droplets on a PDMS surface.

**Figure 1:**
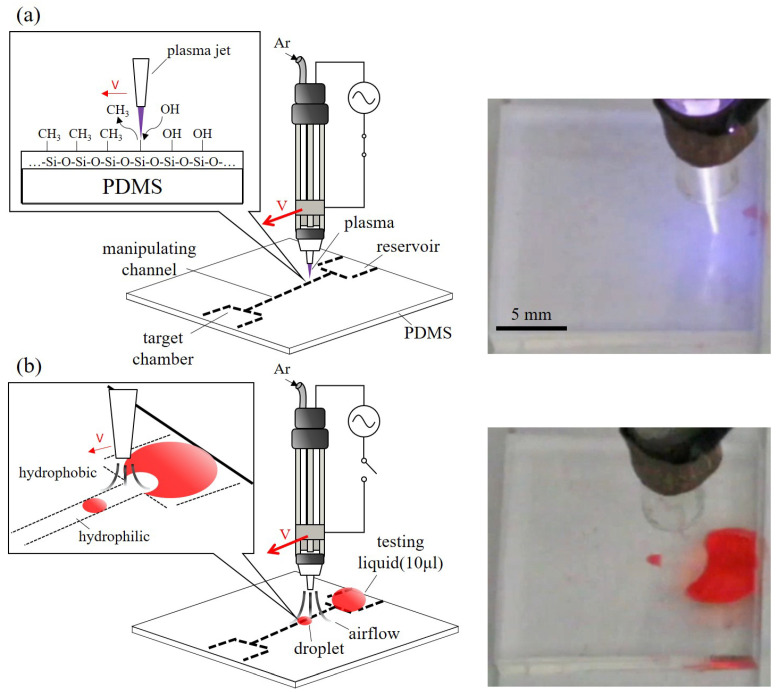
An overview of the proposed method. (**a**) The process of surface modification by plasma jet. (**b**) The process of droplet generation and manipulation by airflow.

Figure 1 shows an overview of the proposed manipulation method. A universal plasma jet is used for manipulating droplets on a PDMS surface. The key technique of the proposed method is to modify the specified area on the substrate for creating a low surface tension area, and use the area as patterned paths for droplet manipulation. The jet has two modes, which are plasma mode and air-jet mode as shown in Figure 1a,b, respectively. During the plasma mode, the jet works as an APPJ, where the electrodes on the jet create an electric field for ionizing the flow-in argon into its plasma state. The ionized plasma is used for modifying the PDMS surface at the nozzle outlet for reducing the surface tension. Since the size of the jet nozzle is finite, the treatment can be applied to only specified areas. As illustrated in Figure 1a, the treated area becomes hydrophilic is due to additional hydroxyl groups (–OH) binding to the PDMS surface while the untreated area remains hydrophobic. The change of wettability is caused by replacing the methyl groups (–CH_3_) of PDMS with the silanol groups (Si–O–H) in the plasma-treated area [41,42]. Different shapes of treated areas can be patterned by the trajectory of the jet moving path, and the width of the treatment can be adjusted by the jet speed. A photo of the plasma generated from the jet in the plasma mode is shown on the right of Figure 1a.

Figure 1b shows the universal plasma jet in the air-jet mode. The power applied to the electrodes on the jet is turned off and results in no electric field for ionizing the argon gas. The argon is flowed through the jet from the inlet to the outlet and create a force field by the jetted gas. The liquid on the PDMS surface is driven by a drag force from the force field for the droplet manipulation, which includes separating and moving droplets. A snapshot of the universal jet moving a droplet is shown on the right of Figure 1b.

To the best of the authors’ knowledge, this is the first work using a universal plasma jet to generate and manipulate droplets on a PDMS surface. The proposed method aims to provide a convenient, controllable, and low-cost way to manipulate droplets for studies in different fields, such as cell culture with droplets encapsulating living cells. Although conventional methods often take a long time and require well-trained skill to fabricate a platform for droplet manipulation, the proposed method can directly generate and manipulate droplets on an untreated PDMS surface using a universal plasma jet. The proposed method can simplify droplet manipulation on a polymeric surface towards wall-less scaffolds for tissue engineering.

The rest of the paper is organized as follows. The working principles, experimental setup, and procedures are explained in Section 2. The experimental results on droplet manipulation and size control are presented in Section 3. The discussions regarding the potentials and limitations about the proposed method are presented in Section 4. Finally, the concluding remarks are summarized in Section 5.

## 2. Method

### 2.1. Experimental Setup

**Figure 2:**
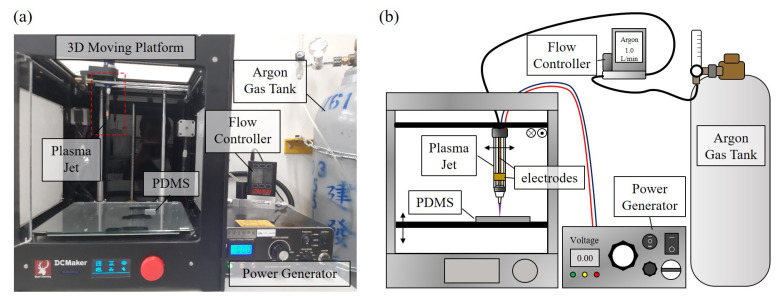
The experimental setup. (**a**) A photo of the experimental setup. (**b**) A illustrative diagram of the experimental setup.

Figure 2a,b show the experimental setup of the system and an illustrative diagram of the setup, respectively. The system is constructed by a universal plasma jet, a 3D moving platform, a flow controller (Alicat Scientific, Tucson, AR, USA, MCM-5LPM-D), an AC power supply (Biowater Technology Inc., Tonsberg, Norway, LTP-A818F), a tank of argon gas (Jian Fang Gases Cor., Zhubei, Taiwan) and a PDMS chip. The flow controller is used to regulate the flow rate of argon which is all set at 1 litre/min in this paper. The power supply is set to output an AC power of 4.5 kV at the frequency of 24 kHz. The power supply is connected to the two electrodes on the universal plasma jet. One is a needle-like electrode along the central axis of the jet while the other is a ring-shaped electrode covering the exterior of the jet using aluminum tape. An electric field is generated between the two electrodes for ionizing bypass gas when the power is turned on. The plasma along with the gas is jetted from the jet nozzle onto the surface. The gap between the outlet of the jet and PDMS chip is 3 mm, and the inner diameter of the jet nozzle is 600 µm. The plasma jet and a PDMS chip are fixed on the 3D moving platform. Standard G-code is used to program the 3D moving platform for controlling the speed and path of the jet.

The property of PDMS surface with and without the plasma treatment is also investigated using Fourier-transform infrared spectroscopy (FTIR) with an FTIR spectrometer (NicoletiS50, ThermalFisher Scientific Inc., Waltham, MA, USA). The scan number for each test is set 64 with the data spacing of 0.121 cm${}^{-1}$. The range of spectrum is set between 4000 and 650 cm${}^{-1}$.

### 2.2. PDMS for Manipulation Platform

PDMS is a kind of polymer that is often used for microfluidic chips because of its biocompatibility and reproducibility [43]. The droplet manipulation in this work is performed using water on a PDMS chip aiming for future cell culture with wall-less scaffolds. PDMS, in general, comprises repeated units of –O–Si (CH${}_{3}$)${}_{2}$. After the plasma treatment, silanol groups Si–O–H substitute for methyl groups –CH${}_{3}$ [42]. Due to the existence of the silanol groups, the surface becomes hydrophilic, and it can be observed by the change of the contact angle [44]. There are many applications of this principle such as PDMS-glass bonding [45], pump-less surfaced microfluidics [46], hydrophilic patterning [47] and etc.

### 2.3. Drag Force for Droplet Manipulation

The motion of a droplet is driven by jetted air from the universal plasma jet in the air-jet mode. The jetted air pushes the droplet to create an unbalance of forces on the droplet and result in its motion or deformation. In order to let the droplet move along a specified path, the surface, where the droplet is, is first modified by the universal plasma jet in the plasma mode, as explained in Section 2.2. The surface with and without plasma modification would have low surface tension and high surface tension, respectively. When a droplet is pushed by the jetted air, the droplet would move along the path of low surface tension because the low surface tension area has less resistance against droplet motion.

**Figure 3:**
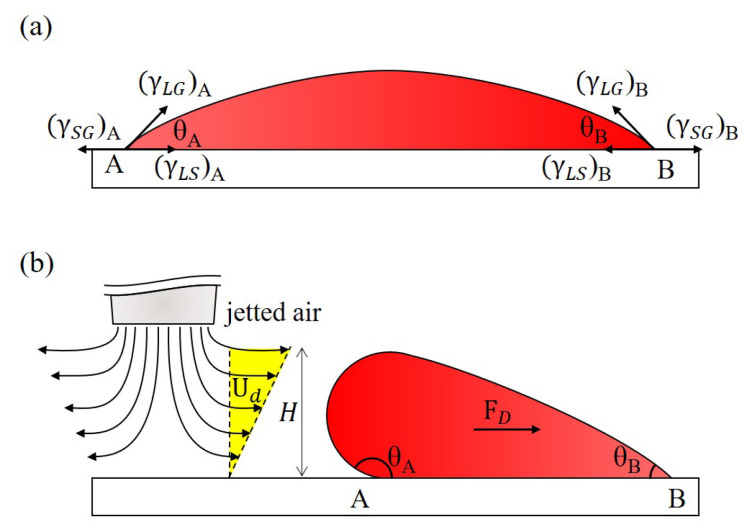
Surface tensions and drag force on a droplet. (**a**) A droplet sits on a surface without jetted air. (**b**) The droplet is deformed and moved by the drag force $F_{D}$ from the jetted air.

Figure 3a shows a droplet sitting on a surface. The contact angles, $\theta_{A}$ and $\theta_{B}$, are determined by surface tensions, as the black arrows shown in Figure 3a, on three interfaces, which are the interfaces between any two of liquid, gas, and solid phases. The balance of the surface tensions on the three interfaces can be formulated by Young’s law as [48]

(1) $${\left(\gamma_{LG}\right)}_{i}\cos\theta_{i}={\left(\gamma_{SG}\right)}_{i}-{\left(\gamma_{LS}\right)}_{i}\;i=A\;\mathrm{or}\;B$$

where $\gamma_{LG}$, $\gamma_{LS}$, $\gamma_{SG}$ and θ are the surface tensions of a droplet on the liquid-gas, liquid-solid, solid-gas interfaces and the contact angle of the droplet, respectively. *A* and *B* are the joint points of the three surface tensions. When the forces on the droplet are balanced, the forces at the points *A* and *B* are cancelled each other and the droplet sits still on the surface.

Figure 3b shows a diagram of the droplet when jetted air is applied to it from the left. Response of the droplet is determined by all forces exerted on the droplet and the forces include drag force from the jetted air, interfacial forces from surface tension and adhesion, buoyant force and inertial force. Among these forces, drag force, $F_{D}$, is the most critical in the proposed work as it is directly used to move the droplet. To simplify the modeling, the shape of the droplet is approximated as a sphere and the drag force $F_{D}$ can be formulated as [49,50,51]

(2) $$F_{D}=k_{x}(6\pi \frac{H}{2}\mu U_{d})$$

where $k_{x}$, *H*, μ and $U_{d}$ are the wall correction factor, the effective height of the droplet, the gas viscosity and the nominal flow speed of the gas, respectively. The wall correction factor is based on the contact between the droplet and the wall. For example, the wall correction factor is 1.7 for a single sphere touching an impermeable wall within simple shear [52].

There are two phases of manipulating a droplet when the jetted air is applied on the droplet. At the beginning, the position of the droplet is held still due to the forces, such as interfacial and inertial forces, adhering the droplet to the surface. The shape of the droplet is consequently deformed by the drag force. The deformation gradually increase the droplet height, *H*, and decrease the contact area on liquid-solid interface. The drag force on the droplet increases with the increase of droplet height, according to Equation (2), while the attaching force to the surface is reduced with the decrease of contact area. Therefore, the drag force and interfacial force on the droplet would keep increasing and decreasing, respectively. The droplet would be eventually moved by the drag force when the interfacial force is no longer able to sustain the droplet at the position. In other words, the droplet is moved by the jetted air when the drag force is greater than all the other forces that prevent the droplet from moving, and the motion of the droplet can be programmed with proper control of the drag force from the jetted air.

### 2.4. Experimental Procedure

#### 2.4.1. Fabrication of PDMS Chip

The PDMS chips are prepared as the platform for droplet manipulation. They are fabricated by mixing PDMS pre-polymer and curing agent (Sylgard 184, Dow Corning Inc., Midland, MI, USA) at the weight ratio of 1:10. The mixture is poured into a container with flat bottoms for PDMS chips with flat surfaces. The thickness of the poured PDMS is controlled at 3 mm. The container of PDMS is put into a vacuum chamber for 30 min to remove the bubbles in the PDMS before putting it into a 90 ${}^{\circ }$C oven for 40 min. Finally, the gel-like PDMS is cured as a solid block and is cut for PDMS chips for the platform of droplet manipulation in experiments.

#### 2.4.2. Plotting of Droplet Path

The droplet path is first plotted with the universal plasma jet in plasma mode for creating a path of low surface tension, and the path is used for guiding the direction of droplet movement. In order to create a stable and uniform path for the droplets. The universal plasma jet is mounted on the 3D moving platform, as shown in Figure 2. The path and speed of the jet are controlled by G codes, a standard motion script widely used for path control. The speeds of 60 mm/min and 300 mm/min are set to the jet for plotting start reservoir and target reservoir, respectively. The reservoirs here are hydrophilic areas where the liquid can be stored on the surface of the PDMS chip. The start reservoir is where the source of liquid is placed, and the target reservoir is where the generated droplets are settled for size measurement.

The speed of the jet moving in the region of the droplet path is controlled by the G-code for creating different widths of the wall-less channels. Different sizes of the droplets are expected to be generated on the channels. A total of nine different speeds of the jet motion for plotting different widths of droplet paths are applied in the test, and are, from low to high, 750, 822, 900, 1050, 1200, 1350, 1500, 1800 and 2100 mm/min. A lower speed of jet motion is expected to make a wider channel with a higher degree of hydrophilicity on the chip since the dose of treatment is greater. In order to quantify the dose of the plasma treatment, the inverse of the jet speed is used for representing the effective time of plasma treatment by the following equation.

(3) $$T_{eff}=\frac{1}{u}$$

where $T_{eff}$ is effective time of plasma treatment, and *u* is the speed of the plasma jet. The applied speeds of plasma jet can be converted into effective time of plasma treatment as $T_{eff}=$ 0.08, 0.073, 0.067, 0.057, 0.05, 0.044, 0.04, 0.033 and 0.029 s/mm, from low speed to high speed, respectively. For the convenience of reading, the effective time of plasma treatment is shortened as treatment time for the rest of the paper.

#### 2.4.3. Generation and Manipulation of Droplets

**Figure 4:**
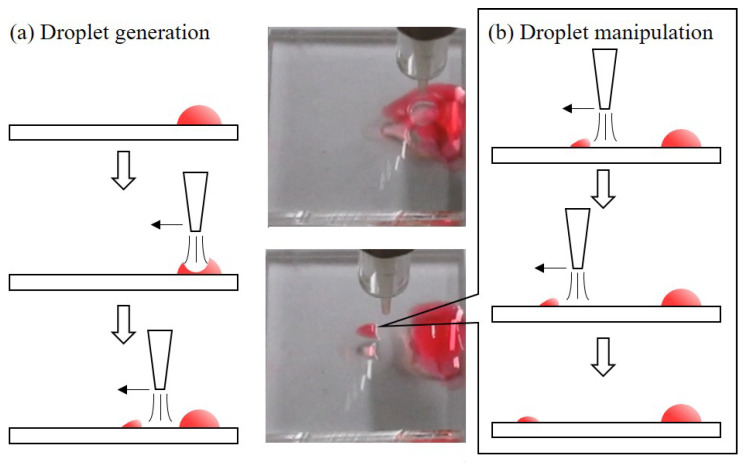
Mechanism of the droplet generation and manipulation with sampled photos from experiments. (**a**) The process of the droplet generation from a source drop. (**b**) The process of the droplet manipulation with jetted air.

Figure 4 shows the steps of droplet generation and manipulation with illustrations and example photos. For the droplet generation, a bulk-size drop is first placed on the plasma-treated path of the surface. The bulk-size drop in the photos is made of colored water with a volume of 10 µL. The universal jet is switch to the air-jet mode with the electric field turned off for generating and manipulating droplets from the bulk-size drop. The argon gas is jetted from the outlet of the jet and generates a force field. When the jet is right above the bulk drop, the force field pushes the surface of the bulk drop against the PDMS surface and creates a circular area as shown in the central-upper photo of Figure 4. With the jet is moving away from the center of the bulk drop, the circular area moves with the jet and at certain points, the jetted gas would separate a small droplet from the bulk drop.

For the droplet manipulation, a drag force from the force field generated by the jetted air is used on the separated droplet, as explained in Section 2.3. The droplet is pushing away from the jetted air. Due to the plasma treatment, the moving direction of the droplet is not random but follows the plasma-treated path by the plasma jet with plasma mode. The speed and direction of the droplet movement is controlled by the jetted air during the droplet manipulation. According to Equation (2), the selection of gas and different flow speed would result in different drag force and different droplet movement.

### 2.5. Measurement of Droplet Size

**Figure 5:**
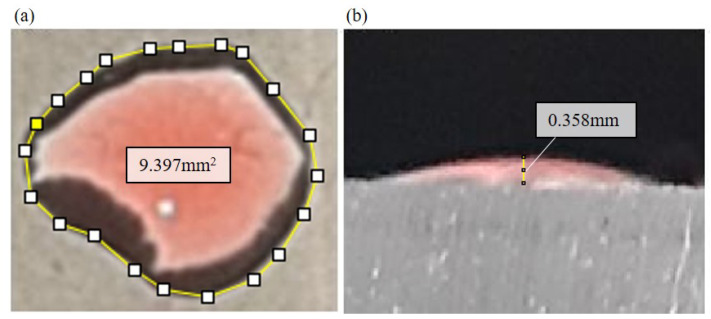
An example of droplet images taken by the top and the side cameras. The size of the droplet is estimated by the covering area and the height. (**a**) Top-view of a droplet. (**b**) Side-view of the droplet.

When the droplet reached the target reservoir, the size of droplet is estimated from images taken from the top and the side of the droplet. The covering area and the height of the droplet are measured using image processing software ImageJ from the top-view and side-view images, respectively. Since the contact angle of the droplet in the target reservoir is small, the size of the droplet is approximated by

(4) $$V=\frac{1}{3}A_{d}h_{d}$$

where *V*, $A_{d}$ and $h_{d}$ are the estimated size, the covering area and the height of the droplet, respectively. Figure 5 shows an example of the size measurement. Figure 5a,b are the top-view and side-view of the droplet. According to the measurement of ImageJ, the covering area and the height of the droplet in Figure 5 are 9.397 mm${}^{2}$ and 0.358 mm. The estimated size is calculated as 1.121 µL. Each test with a different treatment time is repeated at least four times to calculate the average and standard deviation for clarifying the relation of the droplet sizes and treatment time of plasma.

## 3. Results

### 3.1. Effect of Plasma Treatment Time to Droplet Size

**Figure 6:**
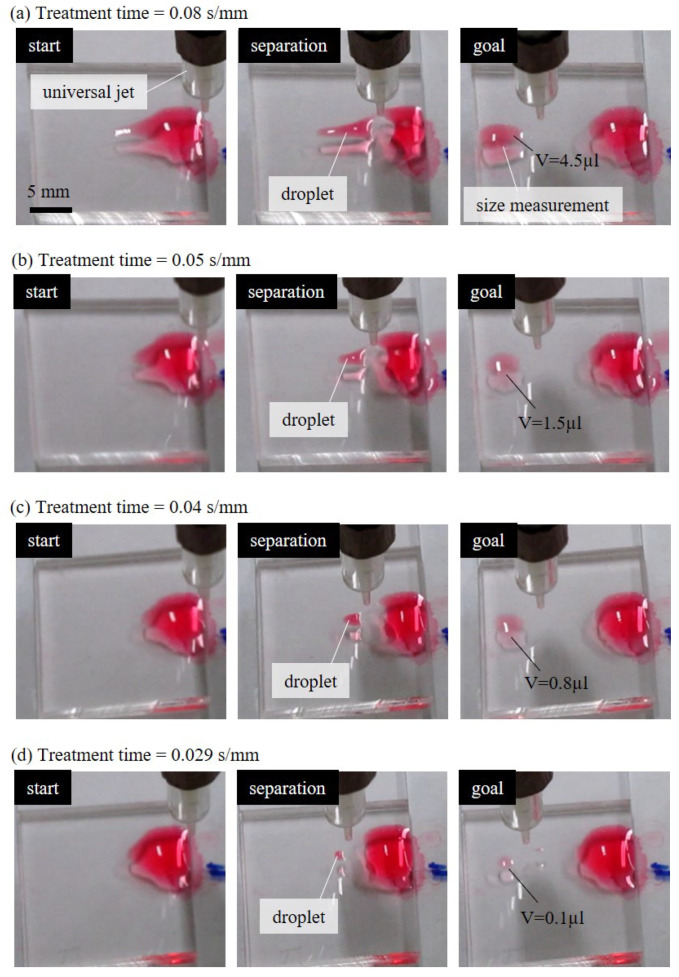
Examples of different size droplets generated and manipulated by different treatment times of plasma. The treatment times are (**a**) 0.08 s/mm. (**b**) 0.05 s/mm. (**c**) 0.04 s/mm. (**d**) 0.029 s/mm.

Figure 6 shows four examples of the experimental results of generating different sizes of droplets with different jet speeds. Figure 6a–d are the generation results with selected treatment times of 0.08, 0.05, 0.04 and 0.029 s/mm, respectively. According to the photos, a longer treatment time resulted in a bigger droplet separated from the source drop. It can be interpreted as that the longer treatment time leads to a greater dose of plasma treatment on the PDMS surface, as well as wider hydrophilic path.

The universal jet in each row of Figure 6 is in air-jet mode and is moved from the right to the left at a constant speed of 300 mm/min.

The initial volume of the bulk-size drop on the right of the PDMS chip is 10 µL. In the case of the longest treatment time of 0.08 s/mm shown in Figure 6a, a big portion from the bulk drop is pushed to the left as the jet moving to the left. When the separated drop reached to the target area and settled, as the “goal” labeled in the rightmost photo of Figure 6a, the size of the separated drop is measured based on top-view and side-view cameras. The size of the pushed-out droplet is getting smaller with the decrease of treatment time, and the smallest droplet is generated with the shortest treatment time of 0.029 s/mm, as shown in Figure 6d. The treatment time is about 3 times shorter while the droplet size is more than 40 times smaller.

**Figure 7:**
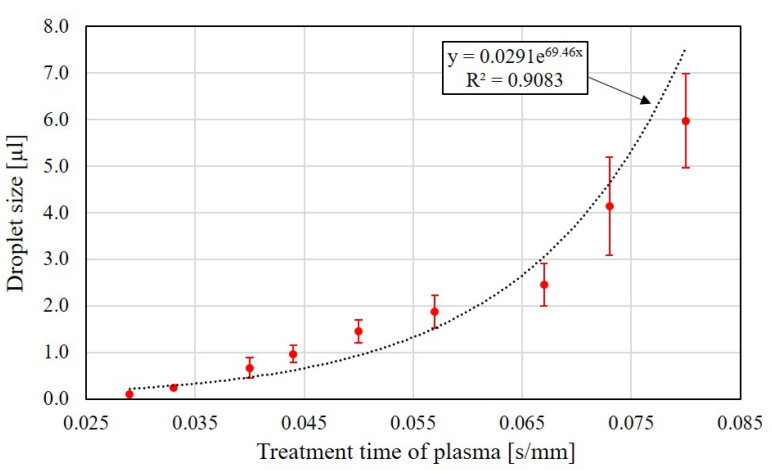
The relation of droplet sizes and treatment time of plasma.

Figure 7 shows the results of all 9 different treatment times and their resulting sizes of droplets during the generation test. Each measurement is repeated at least four times for the 9 different treatment times. The data points and the length of the error bars represent the average and standard deviation of the measured results. A trend of droplet size increasing with the increase of treatment time can be observed. The largest and the smallest sizes of the manipulated droplets are around 6 µL and 0.1 µL, respectively. An exponential function is fit with the points of average values and the correlation coefficient *R* is 0.9083. It shows that the size of the droplet can be controlled by different jet moving speeds, i.e., a smaller size droplet can be generated with a faster-moving speed and vice versa. On the other hand, the standard deviations are found increasing with the increase of the treatment time, and it indicates that the generation of droplet size requires additional control of manipulation conditions for big droplets.

### 3.2. Droplet Mixing with Controllable Droplet

**Figure 8:**
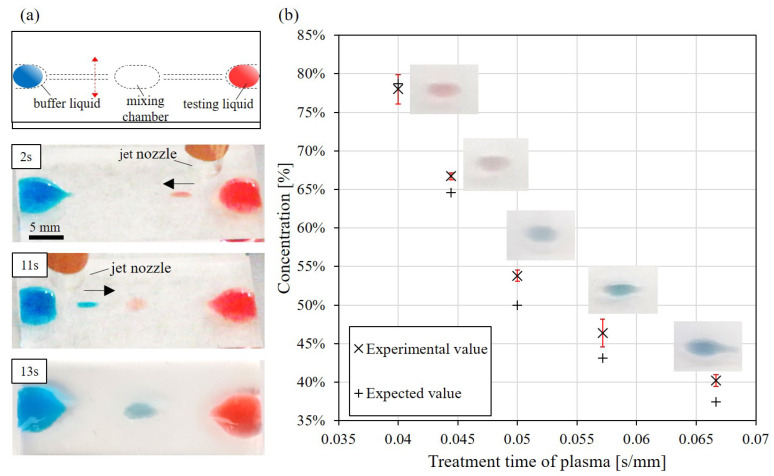
Mixing test. (**a**) The mixing system and the processes of the mixing test. (**b**) Comparison of experimental values and expected values in the mixing test.

Figure 8 shows the application of the proposed method for performing a mixing task on a PDMS chip. Figure 8a shows the design of the mixing chip and snapshots of the mixing process during the test. Two different liquids of the same volume of 10 µL are first placed on two sides of the chip, as the blue and red-colored water shown in Figure 8a. The blue and red-colored water are labeled as buffer liquid and testing liquid in Figure 8a, respectively. The dashed lines in the top diagram of Figure 8a indicate the path of plasma treatment. The center of the chip is where the two liquids are mixed. The snapshots in Figure 8a demonstrate how the two liquids are moved together for the mixing. The red-colored liquid is first moved to the center and is followed by moving blue-colored liquid to the center. The operation is designed as a concentration control in droplet-based applications [53].

Figure 8b shows the results from the mixing test. For controlling the concentration of the final mixed liquid, five different treatment times of plasma from the side of buffer liquid, the blue one, to the center are applied. The treatment times for the path to the buffer liquid includes 0.040 s/mm, 0.044 s/mm, 0.050 s/mm, 0.057 s/mm and 0.067 s/mm. On the other hand, the treatment time for the path to the testing liquid, the red one, is fixed at 0.050 s/mm. The mixing test with each treatment time is repeated four times. The experimental value of the concentration is determined based on the volume ratio of the droplets from two sides, i.e., when a droplet is separated from the source drop, the size and height are visually measured before it reached the center chamber. The expected values are predicted from the relation of droplet size and treatment time of plasma from previous results in Figure 7. According to the comparison in Figure 8b, the differences between experimental values and expected values are 3%, 3%, 4%, 2% and 1% for the treatment times of 0.04, 0.044, 0.05, 0.057 and 0.067 s/mm, respectively. It demonstrates the repeatability and feasibility of the method for mixing applications.

### 3.3. FTIR Spectrum on PDMS Surface

**Figure 9:**
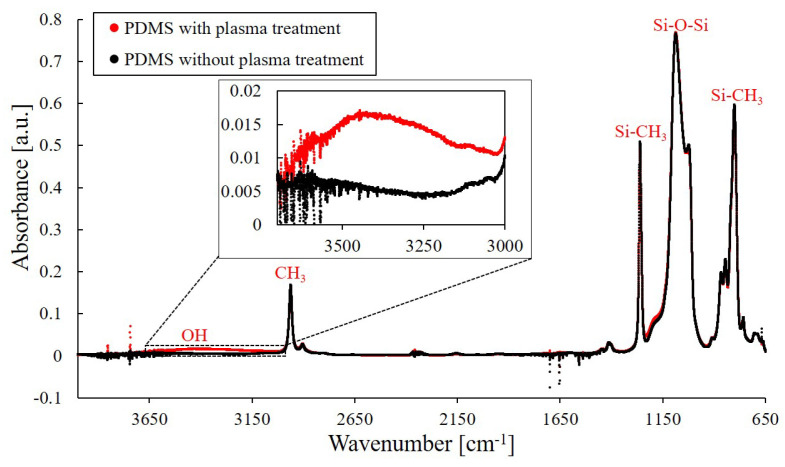
The spectra of PDMS surface with and without plasma treatment is measured with a FTIR spectrometer. A weak broad peak is observed in the range of 3000 and 3700 cm${}^{-1}$.

The treatment of argon plasma on the PDMS surface is investigated using a FTIR spectrometer, and the results are shown in Figure 9. The red and black data in Figure 9 represent the obtained spectra of the PDMS surfaces with and without plasma treatment, respectively. For the convenience of reading, potential functional groups, such as OH and CH${}_{3}$, are labeled in Figure 9 according to the infrared spectrum table (IR Spectrum Table by Frequency Range, Merck KGaA, Darmstadt, Germany). FTIR Scanning is performed 64 times during a single measurement to enhance the scanning results. The results show that the spectra of PDMS surface with and without plasma treatment are mostly overlapped with each other. A weak broad peak is found around the wavenumber between 3000 cm${}^{-1}$ and 3700 cm${}^{-1}$ in the spectrum of the PDMS with plasma treatment. The peak indicates the formation of hydroxyl groups on the PDMS surface upon the plasma treatment, and is well matched to previous results on plasma treatment of PDMS surfaces [54,55,56]. The results show that the plasma treatment with the proposed universal jet enhanced the hydrophilicity of the PDMS surface, and the jet in its plasma mode can be used for plotting paths for the droplet manipulation.

## 4. Discussion

### 4.1. Aging of Plasma-Treated Surface in Atmosphere

**Figure 10:**
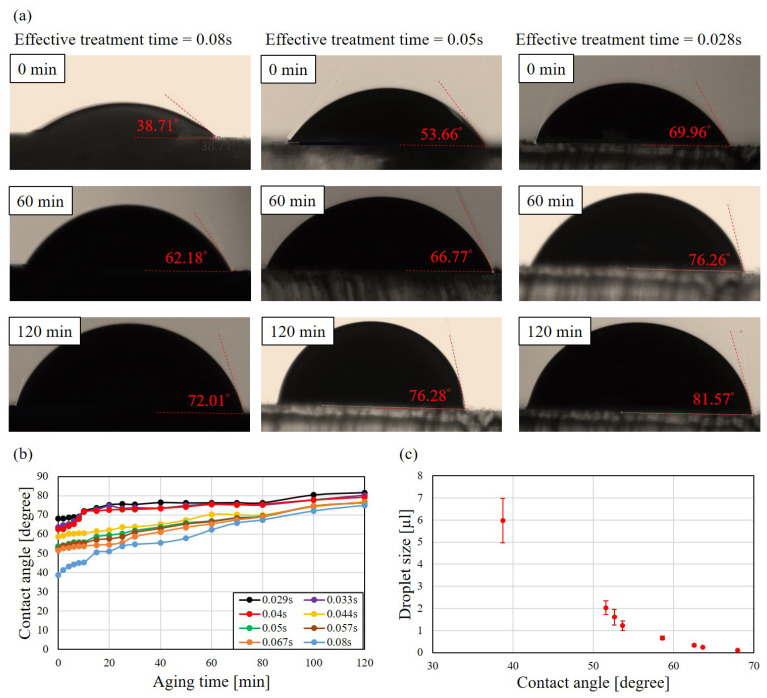
Aging effect of plasma treatment. (**a**) The measurement of contact angle of treatment time of plasma of 0.08, 0.05 and 0.029 s at aging time of 0, 60 and 120 min. (**b**) Aging effect of different treatment time of plasma. (**c**) The relation of contact angle and droplet size.

The sustainability and aging on plasma-treated surface is critical to the proposed method and is discussed here. It is known that th hydroxyl groups on the treated surface are not permanent and the surface would return to hydrophobic condition in an open space [57,58]. Figure 10a shows sample photos of the measurement of contact angles with three different treatment time of plasma. The treatment time, from the left to the right, are of 0.08, 0.05 and 0.029 s while the aging time, from the top to the bottom, are 0, 60 and 120 min, respectively.

At the moment of 0 min, the contact angles are $38.71^{\circ }$, $53.66^{\circ }$ and $69.96^{\circ }$, and it shows that the longer treatment time would result in a smaller contact angle. The contact angles after 60 min are shown in the second row in Figure 10a. the contact angles increase to $62.18^{\circ }$, $66.77^{\circ }$ and $76.26^{\circ }$, from the left to the right, respectively. After two hours, the contact angles increase to $72.01^{\circ }$, $76.28^{\circ }$ and $81.57^{\circ }$.

Figure 10b shows the aging effect of different treatment time of plasma. It shows the trends that the contact angles on the plotted channels become larger with the increased aging time. All the treated surfaces nearly recover to $80^{\circ }$ after 120 min aging time. Figure 10c shows the relation of contact angles and manipulated droplet sizes. Combination of Figure 10b,c, it can be observed that manipulated droplet size becomes smaller with the increased aging time. Therefore, the timing of droplet manipulation with the proposed method is important for repeatability and reliability.

### 4.2. Aging of Plasma-Treated Surface during Droplet Manipulation

**Figure 11:**
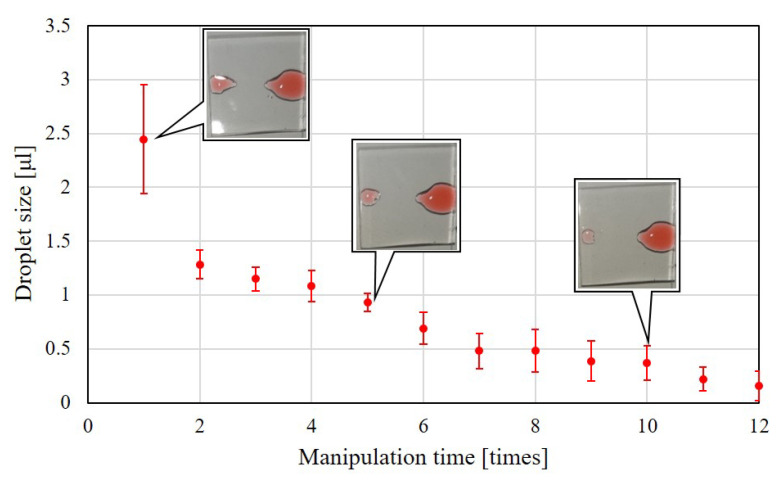
Droplet size is found decreased with the increase number of manipulations on the same plasma-treated path.

The aging effect of the plasma-treated surface is also tested during the droplet manipulation. The aging is expected to be much faster during the droplet manipulation because hydroxyl groups could be easily taken away by the droplet, and particularly, water is used for the droplet in the test. Figure 11 shows the results of droplet size with respect to manipulation time when the effective time of plasma treatment is set to 0.067 s/mm. One time of manipulation means moving a droplet from the liquid reservoir to the target chamber. The time between every two manipulation is kept about one minutes and the liquid reservoir is filled up with 10 µL for the consistency during the test. The marks and error bars in Figure 11 represents the averages and standard deviations of test over 3 times. The results show that the droplet size becomes about half after 5 times of droplet manipulation, which is approximately 4 min after the first manipulation. The photos show the droplet size after the manipulations.

**Figure 12:**
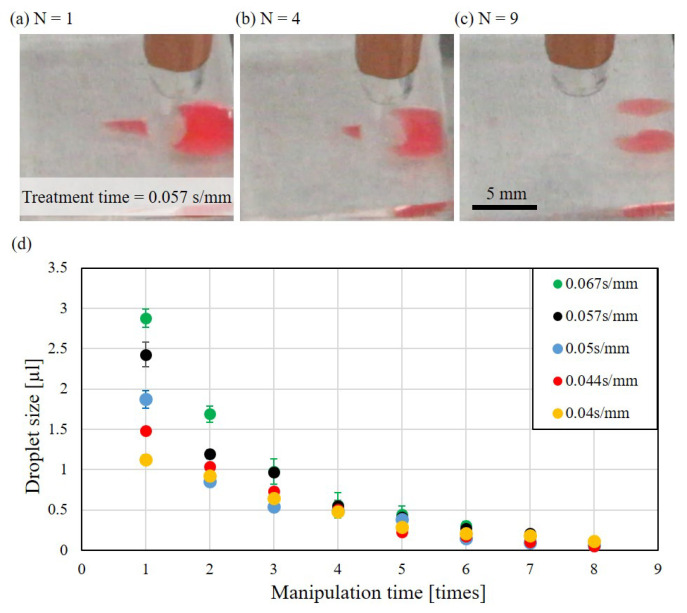
Repeated droplet generation with different plasma treatment. (**a**) A photo of the first droplet. (**b**) A photo of the fourth droplet. (**c**) No droplet is generated after nine times of generation. (**d**) The relation of the droplet size and times of generation at different treatment time of plasma.

The size of the droplet may also depend on the size of the bulk drop in the liquid reservoir and is tested with repeating generations of droplets from the same bulk drop. Figure 12 shows the experimental results of the test. Figure 12a–c are the sample images taken during the processes of manipulation times N of 1, 4, and 9, respectively. The droplet size decreases with the increased number of generations. When the numbers of droplet generation achieved 9, no droplet can be generated from the drop. This could be combined effect of aging of plasma treatment and size of bulk drop.

Figure 12d summarize the same repeating generation of droplets with different treatment times of plasma. A similar trend of the droplet size that the size decreases with the increasing number of droplet generation has been observed among different treatment times of plasma.

### 4.3. The Comparison and Limitations

A table of comparison among different technologies that manipulate droplet are shown in Table 1. Although EWOD and magnetic system are fairly matured for droplet manipulation, the proposed method has great potential that it only needs a few seconds to build up a manipulation platform, which is especially convenient for disposable chips for digital microfluidics. simplicity and easy maintenance are also the advantages of using such a multipurpose plasma jet in a manipulation system. In addition, the plasma jet can apply plasma-generated functional groups to droplets by turning on the electric field on the jet while handling the droplets with the jet. It may provide a useful assist to cell adhesion and cultivation in potential applications. For example, the sterilization function of low-temperature plasma could be a useful assist for handling droplets encapsulating living cells.

Aging, roughness, and differences in surface tension can affect the performance of the manipulation system and are challenges and limitations for the proposed method. Further investigations on these issues are needed for improving the proposed method, or to develop new features based on these limitations. For example, there is a potential for employing the aging effect for re-writable paths of droplets on a PDMS surface for a multifunction platform.

## 5. Conclusions

A novel method for manipulating droplet on a PDMS surface with a universal plasma jet is proposed and tested in this work. The droplets can be generated and manipulated by the plasma mode and air-jet mode of the universal plasma jet. The main concluding remarks are listed as follows.

- The size of droplet can be controlled by the treatment time of plasma using the universal plasma jet. The relation of droplet size and treatment time of plasma has been experimentally clarified. It shows that shorter treatment time of plasma results in smaller size of manipulated droplet. The largest and the smallest sizes of manipulated droplet are around 6 µL and 0.1 µL, which are achieved by the treatment time of 0.08 s/mm and 0.029 s/mm, respectively.
- An application of mixing two colored liquids has been successfully performed. The difference between the expected concentration and actual concentration are all below 5%, which demonstrates the feasibility of applying the proposed method in an application.
- The aging of the plasma-treated path in both atmosphere and during manipulation have been investigated. The contact angles of channel become larger with the increased aging time and nearly recovers to $80^{\circ }$ after 120 min aging time in every tests of treatment time of plasma. The aging is even faster during the manipulation due to the direct contact with droplets.

## Figures and Tables

**Table 1:** Comparison of droplet manipulating technologies.

	Universal Plasma Jet (Proposed)	Electrowetting on Dielectric (EWOD)	Magnetic-Controlled Droplet
**mechanism**	surface treatment and hydrodynamic force	electrical wetting and pressure gradient	magnetic force on particles or fluid
**droplet size**	submicrolitre to microliters	picolitre to tens of microliters	submicrolitre to tens of microliters
**fabrication time of platform**	few seconds for path plotting	several days for multilayer structure	minutes/hours for surface coating and magnets arrangement
**cost**	low air or selected gas	high multilayer and electrodes fabrication	medium magnetic beads/fluid
**features**	can directly apply on polymeric substrate functional radicals	accurate control no need pumps/valves no need fluid pathways	particles can be used as functional substrate can be manually operated
**Ref**	This work	Nelson and Kim 2012 [59]	Zhang and Nguyen [60]

## Data Availability

Data are available upon reasonable request.

## Abbreviations

The following abbreviations are used in this manuscript:

APPJ	Atmospheric-Pressure Plasma Jet
PDMS	Polydimethylsiloxane
